# Lifestyle interventions addressing cardiometabolic health among Black American women of reproductive age in the U.S. : an integrative review

**DOI:** 10.1186/s12884-025-07490-7

**Published:** 2025-05-19

**Authors:** Eyitayo O. Owolabi, Kougang Anne Mbe, Stephen L. Clancy, Renaisa Anthony, Yuqing Guo

**Affiliations:** 1 Center for Health Promotion and Disease Prevention, Edson College of Nursing and Health Innovation, Arizona State University, Phoenix, AZ USA; 2 Betty Irene Moore School of Nursing, University of California, Davis, Davis, CA USA; 3 Research Librarian for Health Sciences and Nursing Science, Science Library, University of California, Irvine, Irvine, CA USA; 4 Momentum Park, San Jose, CA USA; 5 Sue & Bill Gross School of Nursing, University of California, Irvine, Irvine, CA USA

**Keywords:** Cardiometabolic, Lifestyle, Black American, Pregnancy, Postpartum, Pre-Pregnancy

## Abstract

**Background:**

Cardiometabolic disorders among childbearing women, particularly Black American women, contribute to adverse perinatal outcomes and long-term health consequences. Lifestyle interventions are critical approaches to improve cardiometabolic health.

**Objective:**

This integrative review synthesized evidence on lifestyle interventions promoting cardiometabolic health among Black women of childbearing age in the U.S.

**Methods:**

A comprehensive search strategy was developed and applied across PubMed, CINAHL, the Web of Science Core Collection, and Scopus from the databases’ inception through August 2023. Key inclusion criteria were Black American women of childbearing age, lifestyle interventions using an experimental/quasi-experimental design conducted in the U.S., and cardiometabolic, health behavior, or psychosocial outcomes.

**Results:**

Thirty-three studies were included, with 29 (87%) using randomized controlled trials. Lifestyle interventions were primarily implemented during pregnancy and/or postpartum periods, only two in pre-pregnancy stage. Health education (i.e. structured/unstructured teaching on various lifestyle content) was the main intervention component. While many studies incorporated digital health technologies, only six leveraged mhealth tools (e.g., mobile health applications, internet-based platforms, social media) as the primary delivery method. Weight change was the most common cardiometabolic outcome, with five out of 13 studies showing significant reductions in gestational weight gain or postpartum weight retention. Of seven studies measuring other cardiometabolic outcomes (e.g., blood glucose), only one showed a significantly decreased incidence of hypertension. Three of 11 studies reported a significant increase in physical activity, and four out of ten showed significant improvement in dietary behaviors. Nine of the 15 studies measuring psychosocial outcomes found significant improvement, with five noting decreased depression. Common weaknesses included recruitment challenges, convenience sampling, small sample sizes, high attrition rates, and short post-intervention follow-up. Some studies adopting digital health technologies reported better retention rates and higher engagement.

**Conclusions:**

The results suggest the potential impact of lifestyle interventions on weight reduction, increased physical activity, healthier dietary behaviors, and decreased depression. Future high-quality and powered studies are needed to investigate the efficacy of lifestyle interventions on cardiometabolic outcomes in this population by considering the use of digital health technologies to improve intervention recruitment, engagement and retention, including Black American women of childbearing age representing all socioeconomic levels, and targeting the pre-pregnancy stage.

**Supplementary Information:**

The online version contains supplementary material available at 10.1186/s12884-025-07490-7.

## Introduction

Cardiometabolic disorders remain a major public health challenge in the United States (U.S.) [1]. These disorders encompass various diseases affecting the endocrine and cardiovascular systems. The most prevalent among them include hypertension and diabetes mellitus (DM), as well as their pregnancy-specific variants such as hypertensive disorders of pregnancy and gestational diabetes mellitus (GDM) [1]. Cardiometabolic disorders among childbearing women (ages 16–49) [2] create adverse clinical outcomes such as preeclampsia, polyhydramnios, cesarean delivery, preterm births, low birth weight, and large for gestational age neonates [1, 3, 4]. Furthermore, these disorders have long-term effects on both mother and child’s cardiometabolic health [5, 6]. Black American women have a disproportionately higher prevalence of cardiometabolic disorders, estimated at approximately 24% [7] and 57% [7, 8] for DM and hypertension, respectively, compared to 22% and 50% among non-Hispanic White (NHWs) women. In addition, the prevalence of overweight or obesity among Black American women of childbearing age is 64.1% compared to 44.2% among NHW [9]. Thus, developing and implementing effective strategies to promote cardiometabolic health in this high-risk population is crucial. 

In addition to environmental and structural factors [10], several behavioral risk factors (e.g., physical inactivity, unhealthy diets, inadequate sleep, smoking, and alcohol use) [11] and psychosocial risks (e.g., stress, depression, and anxiety) contribute significantly to the development of cardiometabolic disorders [12–14]. Black American women bear a disproportionately higher burden of these risk factors. Excessive prenatal weight gain and postpartum weight retention contribute to long-term overweight and obesity among women of childbearing age [15]. Black American women of childbearing age also have a higher prevalence of diagnosed sleep disorders (8.2%) compared to NHW (2.9%) and are least likely to meet their physical activity recommendation, with a rate of 17% compared to 24% among NHW [16]. To promote maternal health equity, it is imperative to design and deliver targeted interventions that mitigate cardiometabolic risk factors among Black American women.

Lifestyle interventions to improve the cardiometabolic health of Black American women of childbearing age are essential for optimal neonatal and maternal cardiometabolic outcomes and long-term health. Existing literature describes various interventions aimed at improving childbearing-age women’s cardiometabolic health, which include nutrition and physical activity education, lifestyle coaching, pregnancy weight gain, breastfeeding, and childcare [17, 18]. The timing of the interventions ranges from the pre-pregnancy to the postpartum periods. Despite an increased number of studies, evidence of the impact of lifestyle interventions on the cardiometabolic health of Black American women of childbearing age is scant. This integrative review sought to synthesize existing evidence on interventions aimed at addressing cardiometabolic health among Black American women of childbearing age in the U.S. Findings from this review can inform future interventions focused on reducing these risk factors among Black American women of childbearing age to improve their cardiometabolic health.

## Methods

This integrative review was conducted following the Joanna Briggs Institute (JBI) evidence synthesis guideline for review [19], which comprises five phases: defining the research question, identifying relevant studies, selecting the studies, extracting the data, and synthesizing and reporting the findings.

### Research question

This study aimed to synthesize existing evidence on interventions designed to improve cardiometabolic health among Black American women of childbearing age. We sought to describe the timing of interventions (pre-pregnancy, pregnancy, postpartum), the components of these interventions, and reported outcomes. The specific research question was: What is the current evidence of interventions aimed at improving the cardiometabolic health of Black American women of childbearing age?

### Search strategy

Potentially relevant studies were retrieved from four online academic databases using a comprehensive search strategy developed by a health sciences librarian (SC) with input from the study team (EOO, YG, AM): PubMed (U.S. National Library of Medicine/NCBI), CINAHL Complete (EBSCOhost), the Web of Science Core Collection (Clarivate), and Scopus (Elsevier). Databases were searched from inception to August 2023, when the search was completed. Search strategies used a combination of keywords and subject headings as supported by each database. Due to some keywords’ non-specific and ubiquitous nature, specific field designations were added where necessary.

Searches were initially executed on June 12, 2023, in PubMed and CINAHL Complete. Additional searches were performed on August 2, 2023, in the Web of Science Core Collection and Scopus. Each set of search results was imported into the Covidence Systematic Review Software (Veritas Health Innovation Ltd, Melbourne, Australia) [20]. Search results were limited to English-language publications. Keywords and filters were applied to all search strategies (except Scopus, due to the small number of results) to focus on specific study types such as clinical trials, pilot projects and studies, controlled trials, experimental or quasi-experimental interventions, and feasibility studies. Search strategies are summarized in Supplementary Table 1.

### Eligibility criteria

To be eligible for inclusion, studies were required to meet the following criteria: (1) involve Black American women of childbearing age or include at least 30% Black American women if the sample had mixed racial/ethnic groups (2) adopt an experimental or quasi-experimental design, (3) involve lifestyle interventions aimed at changing one or more health behaviors, (4) measure cardiometabolic, health behavior, or psychosocial outcomes (Table 1), (5) be conducted in the U.S., and (6) be written in English. Given the scoping nature of the review, the 30% Black American representation threshold was selected to promote inclusivity. A similar threshold has been previously used in reviews focusing on Black American women [21].

**Table 1:** Outcomes of interest

Cardiometabolic outcomes	Health behavior outcomes	Psychosocial outcomes
Overweight/obesity, weight gain/retention, glycemic control/HbA1c/blood glucose, blood pressure, gestational hypertension, preeclampsia, gestational diabetes, heart rate, lipid profile, inflammatory factors such as interleukin	physical activity, diet, sleep, smoking	Stress, depression, anxiety, social support, self-efficacy, and quality of life

### Study selection

Articles retrieved from the online databases were imported into the Covidence Systematic Review Software [20], and duplicates were removed. The titles and abstracts of the studies were screened independently by two reviewers, followed by a full-text screening using the same protocol. A third reviewer resolved any conflicts arising during the screening process.

### Data extraction

Two independent reviewers extracted relevant data from each included study to enhance rigor. Data extraction was performed using a Microsoft Excel form that was pilot-tested by the authors using a few sample articles and refined accordingly. The extracted data included the following study details: title, first authors, publication year, design, setting, sample size, participant characteristics, intervention components and delivery approach, intervention dosage, cardiometabolic, behavioral, and psychosocial outcomes. Extracted data were summarized descriptively and narratively.

## Results

### Selected studies

**Fig. 1:**
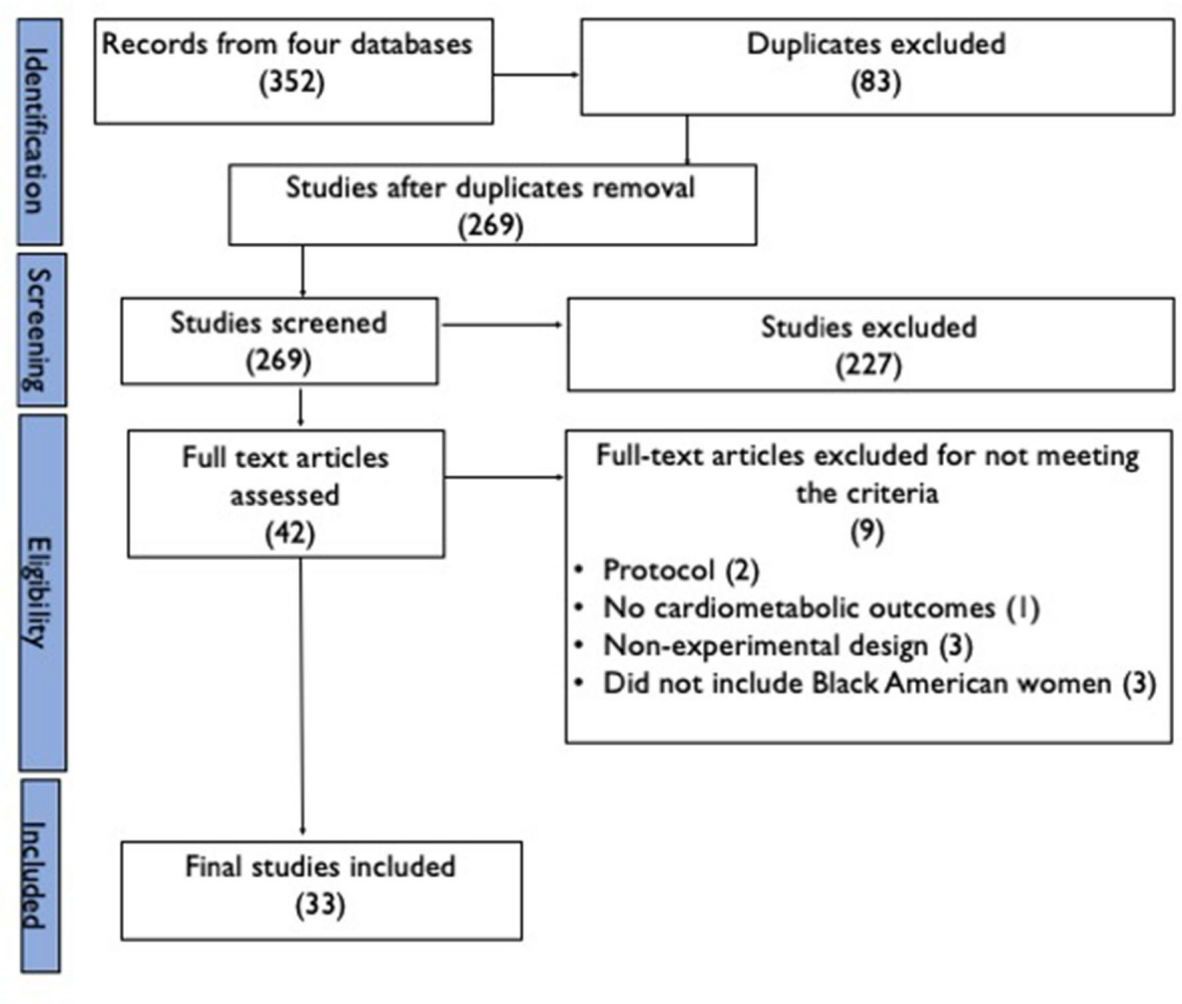
PRISMA flow diagram of the studies screening and selection process

A total of 352 articles were retrieved from the four databases and imported into Covidence. Of these articles, 83 duplicates were identified and removed by Covidence, leaving 269 articles for screening. During the initial title and abstract screening, 227 irrelevant items were excluded. The full-text review excluded nine items for the following reasons: two were protocols, one did not focus on outcomes of interest, three were neither quasi-experimental nor actual experimental studies, and three did not specifically focus on Black American women. Ultimately, 33 studies were included in the final analysis. Figure 1 presents details of the study selection.

### Characteristics of studies

The 33 included studies were published between 1997 and 2023, with a majority, 26 (79%), published between 2012 and 2023. Most studies (29, 87%) utilized a randomized controlled trial (RCT) design, while 4 (12%) used a quasi-experimental design. Twenty-five studies (76%) had a theoretical framework, and 7 (21%) reported that the intervention was culturally tailored. Sample sizes ranged from 16 to 1,139 participants, with an average of 161. The reported mean age of the participants ranged from 18 to 32 years (mean±SD: 27±3). There were only eight studies with 100% Black American women, and the sample sizes ranged from 16 to 913. The remaining 25 (76%) studies included diverse racial participants, with nearly half having less than 70% representation of Black American women.

### Study location distribution

The studies were distributed across five U.S. regions. Sixteen studies (48%) were located in the Southern region, ten (30%) in the Northeastern Region, three (9%) in the Midwestern region, and two (6%) in the Western region. One additional study reported sampling across the Northeast, Midwest, West, and South regions, while another indicated its samples were nationally representative.

### Evidence level

The Johns Hopkins Evidence-Based Practice guideline [22] was used to assess the quality of the articles included in this review. Using this guide, the selected articles were rated for their evidence levels, ranging from 1 (highest evidence level, e.g., RCTs) to 3 (lowest evidence level, e.g., non-experimental study), and for their quality, ranging from high (consistent generalizable results) to low (little evidence with inconsistent results). Most studies (*n* = 29; 88%) were of evidence level 1 (described as studies with experimental or RCT designs) [23–51], while the remaining studies (*n* = 4; 12%) were of evidence level II (comprising studies with quasi-experimental design) [52–55].

### Study quality

Quality assessments were based on three categories: (i) high quality: studies with sufficient sample size, adequate control, consistent and generalizable results, and definitive conclusions; (ii) good quality: studies with sufficient sample size for the study design, some control, reasonably consistent results, and fairly definitive conclusions; (iii) low quality: studies with insufficient sample size for the study design, little evidence with inconsistent results, and non-definitive conclusions drawn. Of the 33 studies, 19 (58%) were rated as low quality [25, 26, 28, 30, 31, 35, 39, 44–55], 12 (36%) were rated as good quality [23, 24, 29, 32, 33, 34, 36, 38, 40–43], and 2 (6%) were rated as high quality [27, 37].

### Stage of intervention across life course

**Fig. 2:**
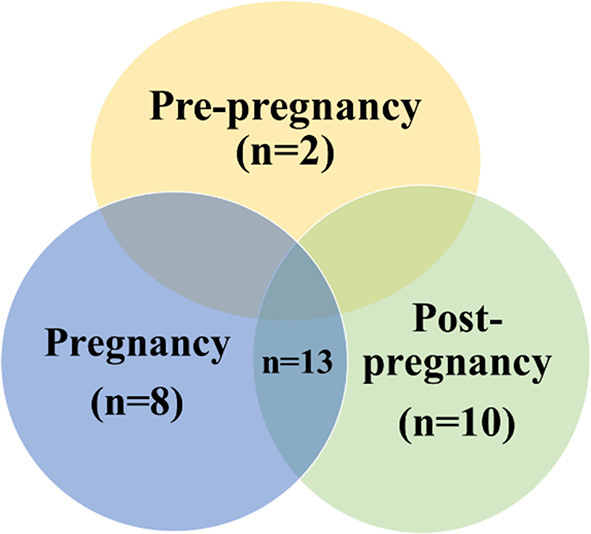
Proportion of Studies Across Life Course

Figure 2 shows that lifestyle interventions for women in these studies were primarily focused on three phases: the combination of the pregnancy and post-pregnancy (postpartum) phases (*n* = 13; 39%) [24, 28, 29, 33, 34, 37, 38, 40, 42, 44, 46, 52, 54], the post-pregnancy phase alone (*n* = 10; 30%) [23, 25, 30, 31, 35, 41, 45, 47, 50, 51], and the pregnancy phase (*n* = 8; 24%) [26, 27, 32, 36, 39, 43, 48, 49]. The interventions were less commonly focused on the pre-pregnancy phase (*n* = 2; 6%) [53, 55].

### Lifestyle intervention

Key Components.

Interventions mainly focused on health education/lifestyle counseling (*n* = 21) [23–26, 30, 31, 33, 34, 37, 40, 42, 44, 46, 48–55] providing evidence-based information and guidance to promote health, prevent disease, and manage health conditions. The most common topics in health education/lifestyle counseling were physical activity (*n* = 16) [23, 30, 31, 33, 35, 40, 42, 45, 47–51, 53–55], nutrition/healthy diets (*n* = 15) [23, 30, 31, 33, 35, 40, 42, 45, 48–51, 53–55], and psychosocial wellbeing such as coping, and stress reduction (*n* = 4) [23, 25, 36, 51]. Less frequently addressed topics included breastfeeding/infant feeding (*n* = 4) [24, 33, 34, 46], childbirth/neonatal and infant care (*n* = 3) [25, 34, 37], sleep (*n* = 3) [25, 33, 35], risky behaviors such as smoking (*n* = 2) [37, 38], and self-care (*n* = 1) [52] (see Supplementary Table 2).

Some interventions included daily to weekly physical activity, diet or weight tracking (*n* = 9) [39, 41–43, 45, 48, 49, 51, 54]. Other interventions (*n* = 7) used cognitive behavioral therapy/psychotherapy [27–29, 32, 36, 44, 46] or social support (*n* = 7) [35, 40, 42–45, 49]. Two studies utilized financial incentives/behavioral economics [39, 41], and another included pharmacologic interventions for risky behaviors such as smoking [38]. The duration of the intervention delivery per session ranged from 20  to 120 minutes. These interventions were mainly conducted weekly or biweekly during the pregnancy phase and monthly in the postpartum phase, lasting approximately between 4 weeks and 12 months (see Supplementary Table 2).

Delivery Approach.

Table 2 illustrates the various intervention delivery approaches: self-monitoring devices (*n* = 12) [23, 26, 35, 39–43, 49, 51, 53, 54], in-person one-on-one education/counseling at a clinic/community center (*n* = 10) [27–30, 32, 38, 42–44, 49], group education/counseling (*n* = 10) [23–25, 28, 31, 36, 48, 50, 51, 54], telephone counseling (*n* = 9) [26, 30, 36, 40, 42–44, 49, 54], in-home visitation (*n* = 7) [33, 34, 36, 37, 47, 48, 50], social media (e.g., Facebook) (*n* = 8) [25, 35, 40, 42, 43, 45, 49, 55], short text messages (*n* = 8) [24, 30, 35, 38, 39, 41, 46, 47], DVD/MP3 player/video disks/podcasts (*n* = 6) [36, 40, 42, 48–50], internet-based platforms (*n* = 3) [41, 46, 55], mobile applications (*n* = 2) [45, 52], or peer mentoring (*n* = 2) [31, 53]. Most studies adopted more than one strategy identified above; 23 studies incorporated digital health technologies [23–26, 30, 35, 36, 38–47, 49, 51–55], with six primarily utilizing mhealth tools (e.g., mobile health applications, internet-based platform, social media) for delivery [25, 41, 45, 46, 52, 55].

**Table 2:** Characteristics of intervention delivery approach

Study	Group education/ counselling	In-person one-on-one education/ counseling	Home visit	Social media	Telephone counseling	Text messaging	Podcast/mp3/DVD	Internet-based platform	Mobile applications	Peer mentoring	Self-monitoring device
Thomson et al. [48]*	X		X				X				
Thomson et al. [50]	X		X				X				
Berry et al. [23]*	X										X
Boyd et al. [25]	X			X							
Crockett et al. [28]	X	X									
Berry et al. [24]*	X					X					
Joshi et al. [31]*	X									X	
Liu et al. [54]*	X				X						X
Walker et al. [51]*	X										X
Liu et al. [42]*		X		X	X		X				X
Wilcox et al. [49]		X		X	X		X				X
Gross et al. [30]*		X			X	X					
Liu et al. [43]*		X		X	X						X
El-Mohandez et al. [29]		X									
Grote et al. [32]		X									
Cinciripini et al. [27]		X									
Kranzler et al. [38]		X				X					
McKee et al. [44]		X			X						
Chao et al. [26]*					X						X
South et al. [47]			X			X					
Jesse et al. [36]	X				X		X				
Haire-Joshu et al. [33]*			X								
Hans et al. [34]			X								
Kitzman et al. [37]*			X								
Herring et al. [35]*				X		X					X
Lane et al. [40]*				X	X		X				X
Napolitano et el. [45]*				X					X		
Lewey et al. [41]						X		X			X
Pezley et al. [46]						X		X			
Bryant et al. [52]									X		
Cavallo et al. [55]*				X				X			
Kannan et al. [53]										X	X
Krukowski et al [39]						X					X

*Included cardiometabolic outcomes

### Study outcomes

Study findings were broadly organized into three categories: cardiometabolic, health behavior, and psychosocial outcomes (see Table 3).

Cardiometabolic Outcomes.

Sixteen unique studies reported on cardiometabolic outcomes. Weight change was the most common cardiometabolic outcome (*n* = 13) [26, 30, 31, 33, 35, 37, 42, 43, 45, 48, 51, 54, 55]. Of these studies, five (38%) reported a significant reduction in excess gestational weight gain or a decrease in postpartum weight retention [30, 33, 35, 42, 43]; the remaining eight (62%) reported no significant difference in weight changes between the intervention and control arm or from the baseline to the post-intervention periods. Among the five studies showing positive impacts, three (60%) had at least 70% representation of Black American women in their study population [30, 33, 35]; the intervention delivery approaches included one-on-one education/counseling delivered in-person or telephonically (*n* =3) [30, 42, 43], text messaging (*n* = 2) [30, 35], and social media (*n* = 2) [35, 42].

Seven studies reported on cardiometabolic disorder-related outcomes: blood pressure (*n* = 3) [24, 33, 40], pregnancy-induced hypertension/pre-eclampsia (*n* = 2) [37, 48], blood glucose (*n* = 2) [24, 33], glucose tolerance (*n* = 2) [24, 26], fasting insulin (*n* = 2) [24, 33], skin folds (*n* = 2) [23, 24], lipids (*n* = 2) [24, 33], Homeostatic Model Assessment for Insulin Resistance (*n* = 1) [33], HbA1 (*n* = 1) [24], waist circumference (*n* = 1) [24], and total cholesterol (*n* = 1) [24]. Only one of these studies reported a significant change between the intervention and control arms, specifically, the incidence of hypertension [37]. Notably, this study, which delivered a home visitation intervention, included participants with 90% being Black American women [37].

Health Behavior Outcomes.

Seventeen unique studies reported on four health behavior outcomes: physical activity (e.g., walking, steps count, time spent in nature; *n* = 11) [24, 35, 39, 41, 43, 45, 47, 49, 50, 53, 55], dietary/nutritional behavior (e.g., fruit and vegetable consumption, fast food consumption, low-fat diet, low sugar diet, calorie intake; *n* = 10) [23, 24, 26, 31, 35, 43, 45, 49, 53, 55], smoking (*n* = 2) [29, 38], and self-monitoring of blood pressure or weight (*n* = 2) [39, 53]. Of the eleven studies that assessed measures of physical activity, only three reported significant changes in the intervention arm compared to control from baseline to post-intervention [24, 41, 45], with one study having ≥ 70% Black American subjects [45]. Among the ten studies reporting on dietary/nutritional behavior, four reported significant improvement [23, 24, 45, 49], with two of these four studies having ≥ 70% Black American representation [23, 45].

Of the seven studies showing significant impact on behavioral outcomes, the intervention delivery methods varied: two used group education/counseling [23, 24], one employed one-on-one education/counseling (*n* = 1) [49], one combined social media with mobile applications [45], and one utilized an internet-based platform [41].

Psychosocial Outcomes.

Fifteen unique studies reported on various psychosocial outcomes, most commonly depression (*n* = 10) [25, 27–29, 32, 34, 36, 44, 46, 52], social support/functioning (*n* = 5) [32, 44, 45, 50, 52], stress/coping/self-efficacy (*n* = 4) [24, 28, 45, 51], anxiety (*n* = 2) [46, 52], resilience (*n* = 2) [28, 52], quality of life (*n* = 1) [49], mother-infant bonding (*n* = 1) [44], and intimate partner violence (*n* = 1) [29]. Of these fifteen studies, nine (60%) reported significant improvement in areas of depression (*n* = 5) [25, 27, 32, 36, 44], coping/self-efficacy (*n* = 2) [28, 51], resilience (*n* = 2) [28, 52], and quality of life (*n* = 1) [49]. Three (33%) of the nine studies that reported significant improvement had ≥ 70% representation of Black American women in the study sample [25, 28, 52].

Of the nine unique studies that reported significant improvement in psychosocial factors, the intervention delivery methods were as follows: three utilized one-on-one education/counseling [27, 32, 44], one involved both group and one-on-one education/counseling [28], four combined education/counseling with social media/podcast/self-monitoring device [25, 36, 49, 51], and one used mobile applications [52].

**Table 3:** Summary of key findings

	Outcomes			
Study	Cardiometabolic	Health Behavior	Psychosocial	%Black
Thomson et al. [48]	**Weight**: No significant difference in the proportion of participants meeting recommended gestational weight gain between the intervention and the control group (*p* = 1.000). **Hypertension and Pre-eclampsia**: No difference in changes in the proportion of hypertension (*p* = 0.399) and pre-eclampsia (*p* = 0.358).	Not Assessed	Not Assessed	95%
Thomson et al. [50]	Not Assessed	**PA**: No significant difference in minutes of physical activity between the intervention and the control group at the three-time points (*p* = 0.075).	**Social Support**: No significant difference in social support between groups (*p* = 0.601).	95%
Berry et al. [23]	**Skinfolds**: Experimental group had significantly greater decreases in triceps skinfolds (*p* = 0.01) and subscapular skinfolds (*p* = 0.04).	**Nutrition Knowledge**: Experimental group had significantly greater nutrition knowledge than the control group (*p* = 0.04).	Not assessed	77%
Boyd et al. [25]	Not Assessed	Not Assessed	**Depression**: Intervention group demonstrated a greater reduction in depression symptoms than the control group (-9.3 vs. -0.1, *p* < 0.01).	84%
Crockett et al. [28]	Not Assessed	Not Assessed	**Postpartum Adjustment**,** Depression, Stress**: At 3 months intervention group reported significantly better postpartum adjustment (13 vs. 10.42, *p* < 0.009), no difference in depression symptoms or parental stress between groups (*p*s > 0.05)	100%
Berry et al. [24]	**WC**,** BMI**,** Skinfold**,** BP HbA1c**,** HOMA-IR**,** Fasting Insulin**,** Fasting Glucose**,** 2-hour Glucose** Compared with the control group, women in the intervention had a significant decrease in waist circumference at 7 months (*p* = 0.046). No significant differences in weight, BMI, skinfolds, BP, total cholesterol, A1C, HOMA-IR, fasting insulin, fasting glucose, and 2-hour glucose (*p*s > 0.05).	**PA**,** Diet**: Compared with control, the intervention group had a significant increase in PA (*p* = 0.033), ate at a fast-food restaurant less (*p* = 0.012).	**Stress Management**: No significant differences in stress management between groups (*p* > 0.05). **Self-Efficacy**: No significant differences in self-efficacy (*p* > 0.05).	52%
Joshi et al. [31]	**Weight**: No difference in weight gain between intervention and control groups (*p* = 0.89).	**Diet**: No significant change in diet behavior: low-fat eating (4.7 ± 2.4 vs. 4.2 ± 1.8, *p* = 0.86).	Not Assessed	100%
Liu et al. [54]	**Weight**: Compared to controls, intervention group were less likely to exceed IOM weight gain recommendations (56.3% vs. 63.2%, *p* = 0.07).	Not Assessed	Not Assessed	100%
Walker et al. [51]	**Weight**: Weight changes between intervention and control groups were non-significant in each ethnic group (*p*s > 0.05).	Not Assessed	**Self-Efficacy, Stress**: African American women’s self-efficacy was a significant psychosocial predictor of weight loss (*r*= -0.48, *p* < 0.05). Lower perceived stress favored weight loss (*r* = 0.48, *p* < 0.05).	35%
Liu et al. [42]	**Weight**: Intervention participants retained less weight than standard care women at 6 months (mean difference– 3.5 kg, *p* < 0.0003) and 12 months (mean difference- 2.2 kg, *p* < 0.03).	Not Assessed	Not Assessed	44%
Wilcox et al. [49]	Not Assessed	**PA**,** Diets**: No change noted in the level of PA. Participants ate more vegetables (0.16 vs.-0.29, *p* = 0.01) and whole grains (8.05 vs. -0.55, *p* < 0.01).	**HRQOL**: Control group had greater improvement in score of HRQOL mental component than the intervention group (4.52 vs. 1.84, *p* < 0.05).	44%
Gross et al. [30]	**Weight**: Intervention participants retained significantly less gestational weight gain at 6-month postpartum than control participants (3.0 ± 11.8 vs. 12.6 ± 20.4, *p* < 0.05). No significant difference in GWG between groups (*p* > 0.05).	Not Assessed	Not Assessed	96%
Liu et al. [43]	**GWG and Percent of Exceeding IOM Guideline**: Black participants with overweight gained 4.5 kg less and had a lower percentage of exceeding IOM guidelines than standard care participants (*p*s < 0.05).	**PA, Diet**: No significant differences in moderate-to-vigorous PA and energy intake between intervention and control groups (*p*s > 0.05).	Not Assessed	44%
El-Mohandez et al. [29]	Not Assessed	**Smoking**: Compared to control group, the intervention group had a greater reduction in environmental tobacco smoke exposure (-301% vs. 25%, *p* = 0.01); and a lower resurgence in smoking rates (2.2% vs. 7.3%, *p* < 0.001).	**Depression, Intimate Partner Violence**: No significant differences in the change for depression and intimate partner violence between the two groups (*p*s > 0.05).	100%
Grote et al. [32]	Not Assessed	Not Assessed	**Depression**,** Social Functioning**: Compared to the control group, intervention group had lower depression diagnosis (5% vs. 42%, *p* < 0.003) and depressive symptoms (24.3 vs. 25.9, *p* < 0.001) Also, the intervention group had greater improvement in social functioning at six months postpartum (3.07 vs. 3.18, *p* = 0.002)	62%
Cinciripini et al. [27]	Not Assessed	Not Assessed	**Depression**: Compared to the control group, the intervention had less depression, at the 6 months after end of treatment (*p* = 0.001).	54%
Kranzler et al. [38]	Not Assessed	**Smoking Abstinence/Cessation**: No difference between groups: At the end of treatment (7-day point prevalence quit rates: bupropion = 11.0% vs. placebo = 18.5%, *p* > 0.05); or week 24 (7-day point prevalence quit rates: bupropion = 9.4%vs. placebo = 21.5%, *p* > 0.05).	Not Assessed	52%
McKee et al. [44]	Not Assessed	Not Assessed	**Depression**,** Social Support**: Depressive symptoms from late pregnancy to 3 months postpartum were significantly reduced in both intervention and control groups (*p*s < 0.05); but no significant group difference in social support and mother-infant bonding (*p*s > 0.05).	43%
Chao et al. [26]	**Weight**,** Glucose Tolerance**: No significant differences in gestational weight (*p* = 0.29), and 1-hour 50 g glucose test between the intervention and control (116.0 ± 35.3 vs. 113.9 ± 23.2 mg/dL, *p* = 0.82).	**Diet**: No significant difference in caloric intake (2,154 ± 251.3 vs. 1,972.0 ± 202.7 kcals; *p* = 0.58).	Not Assessed	54%
South et al. [47]	Not Assessed	**PA**: Women who completed the intervention had three times higher nature visits compared to the control (IRR 3.1, 95%CI 1.16–3.14, *p* = 0.025).	Not Assessed	90%
Jesse et al. [36]	Not Assessed	Not Assessed	**Depression**: The intervention significantly reduced depression scores for African American women at high-risk from baseline to post-treatment (5.59 ± 0.90 vs. 2.18 ± 1.04, *p* = 0.02) and baseline to one-month follow-up (6.32 ± 1.06 vs. 3.14 ± 1.01, *p* = 0.04).	68%
Haire-Joshu et al. [33]	**Weight**: Intervention arm gained less weight (2.5 kg ± 7.4 kg vs. 5.7 ± 8.8 kg; *P* = 0.01) and were more likely to return to to their baseline weight during postpartum (38% vs. 21.5%, *p* = 0.01) compared to control. **BP**,** Glycemic Gontrol**,**Insulin Sensitivity**,** Lipid Profile**: No significant changes between groups (*p*s > 0.05).	Not Assessed	Not Assessed	100%
Hans et al. [34]	Not Assessed	Not Assessed	**Depression**: The change in postpartum depressive symptoms did not differ between the intervention and control groups (p: Not Reported).	45%
Kitzman et al. [37]	**Pregnancy-induced Hypertension**: Fewer women in the intervention group had pregnancy-induced hypertension than the control group (13% vs. 20%; *p* = 0.009). **Weight**: No significant differences in gestational weight gain (*p* > 0.05).	Not Assessed	Not Assessed	90%
Herring et al. [35]	**Weight**: Greater weight loss in the intervention group than usual care: adjusted mean difference: −3.2 kg, *p* = 0.04)	**Diet**: More intervention than control participants endorsed eating “less food” at follow-up (100% vs. 44%, *p* = 0.03). **PA**: No significant difference was found in the number of days spent walking (p: Not Reported).	Not Assessed	78%
Lane et al. [40]	**BP**: The intervention did not reduce the risk of worse systolic or diastolic BP trajectory in overweight/obese women (p: Not Reported).			44%
Napolitano et el. [45]	**Weight**: No significant difference in weight loss between groups *p* > 0.05).	**PA**: No significant difference between groups; but there was a significant increase among the intervention group MVPA (618.4 vs. 279.9 min/weekly; *p* = 0.004) VPA (328.3 vs. 100.1 min/weekly; *p* = 0.001) **Diet**: Intervention groups had significantly higher servings of fruits and vegetables compared to control (*p* = 0.02). No significant difference in fast food consumption between groups (*p* > 0.05).	**Stress**,** Coping**,** Social Support**: No difference between groups (*p*s > 0.05).	100%
Lewey et al. [41]	Not Assessed	**PA**: The intervention group had 647 more steps (95% CI, 169–1124; *p* = 0.009) and increased their step goals (0.47 vs. 0.38, *p*= 0.003) more than the control group.	Not Assessed	55%
Pezley et al. [46]	Not Assessed	Not Assessed	**Depression**,** Anxiety**: Depression and anxiety scores remained below the clinical threshold for referral to treatment in both groups (p: Not Reported).	100%
Bryant et al. [52]	Not Assessed	Not Assessed	**Resilience**,** Depression**,** Anxiety**,** Social Support**: There was a significant improvement in resilience (26.44 ± 5.39 vs.28.29 ± 5.26, *p* < 0.001) but no significant improvement in depression, anxiety or social support (*p*s > 0.05).	99%
Cavallo et al. [55]	**Weight**: At the end of the intervention, the mean weight change among participants was − 1.3 kg ± 4.4 kg (p: Not Reported).	**PA**: Mean change in walking time was 116.3 min/week ± 191.6 (p: Not Reported). **Diet**: The mean change in servings/day of fruit and vegetables was 0.5 ± 1.5 servings/day (p: Not Reported).	Not Assessed	73%
Kannan et al. [53]	Not Assessed	**Diet**,** PA **,** BP Monitoring**: At the end of intervention, 77% adopted at least one healthy eating behavior (moderating sodium, serving more fruits and vegetables to their families); 23% adopted at least two such behaviors (reading food labels for sodium; using culinary herbs/spices; serving more fruits and vegetables to their families); and 45% adopted both dietary (moderating sodium; eating more fruit) and biomedical behaviors such as exercising, BP monitoring (p: Not Reported).	Not Assessed	100%
Krukowski et al.[39]	Not Assessed	**Weight Monitoring**: Those in the weight loss incentive group had 2.86 times as many days of self-weighing as those who received the lottery incentive (*p* < 0.01). **PA**: No difference in physical activity between those who received a physical activity incentive and those who did not (*p* > 0.05).	Not Assessed	62%

WC: Waist Circumference; HOMA-IR Homeostatic Model Assessment for Insulin Resistance; PA: Physical Activity; BP: Blood Pressure; MVPA: Moderate-to-Vigorous Physical Activity; VPA: Vigorous Physical Activity; HRQOL: Health-Related Quality of Life; GWG: Gestational Weight Gain; IOM: Institute of Medicine

### Weaknesses of studies

Various weaknesses were reported in the included studies. The most common were high attrition rates ranging from 25% to 70% (*n* = 17) [23–25, 28, 31, 33, 36, 42, 44–48, 50, 51, 54, 55], and small sample sizes (*n* = 6) [25, 28, 35, 46, 47, 54]. High attrition rates were most commonly reported during the postpartum period, especially for in-person interventions, due to inconvenience for mothers returning to work [24] or women lacking easy access to transportation [23]. Weaknesses such as recruitment challenges (*n* = 3) [42, 43, 49] and convenience sampling (*n* = 3) [27, 43, 46] were also noted. These led to the low representation of people of low socioeconomic status or the recruitment of participants who appeared motivated, potentially introducing bias. Other reported weaknesses included the use of self-reported data (*n* = 3) [25, 28, 50], difficulties in intervention delivery or low app usage for mhealth interventions (*n* = 2) [44, 45], short follow-up/intervention (*n* = 6) [27, 28, 35, 38, 42, 44], and lack of control group (*n* = 3) [52, 53, 55].

## Discussion

Cardiometabolic disorders among women of childbearing age pose significant risks to both maternal and neonatal outcomes, with Black American women bearing a disproportionately higher burden compared to other racial/ethnic groups. This study synthesized available evidence on interventions aimed at improving cardiometabolic health among Black American women of childbearing age. Overall, our review revealed limited evidence of the role of lifestyle intervention on most cardiometabolic outcomes among Black American women of childbearing age, with the exceptions of weight change, physical activities, dietary behaviors, and depression.

This review found that most studies primarily measured weight as the cardiometabolic outcome, with fewer examining other indicators related to cardiometabolic disorders such as diabetes and hypertension. While several studies reported significant improvement in weight [30, 33, 35, 42, 43], only one demonstrated a decreased incidence of hypertension [37]. Notably, the studies showing weight improvements did not measure cardiometabolic outcomes, making it impossible to determine how weight changes might affect important cardiometabolic markers such as blood glucose, HbA1c, and blood pressure [30, 33, 35, 42, 43]. Likewise, the study demonstrating improved cardiometabolic outcome did not measure weight, preventing the establishment of a relationship between this improvement and weight changes [37]. This disconnect highlights the need for a better understanding of mechanisms by which lifestyle interventions impact cardiometabolic health. While weight change is a step in the right direction, given its contributory role to cardiometabolic health, more evidence is needed on effective lifestyle interventions that will significantly improve other cardiometabolic outcomes in Black American women of childbearing age. It is crucial to note that interventions aimed at reducing weight should be approached cautiously, considering the potential for stigmatization. Women in the preconception, pregnancy, and postpartum periods are particularly vulnerable to weight stigma [60], which can adversely affect both physiological and psychological health [56]. Moreover, several studies assess the effect of lifestyle interventions on cardiometabolic-related outcomes indicated by health behaviors and/or psychosocial outcomes. These studies found significant improvements in increased physical activity, healthier dietary behaviors, and decreased depression symptoms [23–25, 27, 32, 36, 41, 44, 45, 49].

Health education/lifestyle counseling emerged as the most common intervention component [23–25, 27–34, 36–38, 42–44, 47–51, 54]. These interventions primarily focused on physical activity and/or diet, with less emphasis on psychosocial well-being or self-care. The three dominant delivery approaches for health education/lifestyle counseling were group formats, home visitations, or in-person individual sessions. Notably, health education was the predominant intervention component in 15 of 22 studies that reported significant improvement in cardiometabolic, health behavior, or psychosocial outcomes. However, the specific mechanism by which health education influenced these improved outcomes remains unclear and warrants further investigation.

While some studies integrated digital health technologies into their intervention delivery strategies, often utilizing methods such as telephone counseling and/or text messaging, few leveraged more advanced mhealth tools such as social media platforms or mobile applications as primary delivery methods. Boyd et al. [25] were one of few studies that adopted a parenting intervention for postpartum mothers with depressive symptoms using a social media platform. Their study revealed a striking difference in attendance rates: 83% for the social media intervention group compared to just 3% for the in-person group. This suggests that digital health technology has great potential to address participant engagement and retention challenges. Future interventions might benefit from increased incorporation of these technological approaches, particularly when targeting Black American women who may face barriers to in-person participation.

Most studies in this review focused on pregnancy and/or postpartum periods, with only two addressing on pre-pregnancy interventions [53, 55]. This finding demonstrates a substantial lack of research aimed at promoting pre-pregnancy cardiometabolic health, especially among Black American women of childbearing age. Despite the limited number of interventions conducted during the pre-pregnancy phase, descriptive studies provide promising evidence that increased preconception cardiometabolic health significantly reduced risks of adverse maternal and neonatal outcomes [57–60]. Given this evidence, there is a clear need to develop and test lifestyle intervention studies focusing on reducing the risks for cardiometabolic disorders among Black American women of reproductive age during the pre-pregnancy phase. The National Institute on Minority Health and Healthy Disparity research framework [61] offers a valuable approach to developing such interventions. This framework emphasizes the importance of adopting a life course approach and considering multilevel and multidomain drivers of health to enhance health equity. Specifically, the life course perspective highlights the cumulative and transmissive effect of various factors on health outcomes. By focusing on the pre-pregnancy period, interventions can reduce risk factors early in the reproductive life course and potentially improve cardiometabolic health during pregnancy and postpartum. Additionally, a multi-dimensional approach can help address the complex factors associated with cardiometabolic health disparities among Black American women by impacting various factors (e.g., individual health behaviors, family dynamics, community resources, or systemic policies). This framework can be utilized to develop more holistic, culturally responsive lifestyle interventions that ameliorate the challenges and meet the needs of pre-pregnancy-aged Black American women in the U.S. [61].

Despite the potential impact of lifestyle interventions on cardiometabolic health (particularly weight change) among Black American women, this review’s findings should be interpreted with caution due to the highlighted weaknesses of the studies included in this review. The most commonly reported weaknesses were small sample sizes, high attrition rates, or low engagement, which compromised the validity of the study findings. For instance, in a study with a high dropout rate, participants preferred telehealth platforms in the postpartum period, reporting the need to return to work and limited time availability [24]. Conversely, a few studies reported low attrition rates or high engagement. One such study, which addressed psychosocial factors of coping and depression, reported high engagement with a mean attendance of 4.58 out of five intervention sessions [28]. However, despite noting transportation challenges among participants, this study did not describe strategies to maintain attendance [28]. Another study attributed high engagement and low attrition to their recruitment strategy, which likely attracted highly motivated participants; however, it failed to report the specific methods employed to increase engagement and retention [27]. Future studies should prioritize larger sample sizes and adopt effective strategies for better patient engagement and retention. Achieving this goal requires considering more flexible and accessible delivery methods. Technology offers a promising method to improve accessibility, enhance adherence to care, and increase continuity of care [62, 63]. Researchers can potentially reduce barriers to accessing care by integrating technological solutions into future interventions, a critical step in addressing health disparities. Additionally, researchers should also endeavor to report their intervention implementation strategies, providing valuable insights for the replication and scaling of successful approaches. Addressing these methodological issues and leveraging technology can produce more rigorous and generalizable findings on lifestyle interventions aimed at improving cardiometabolic health among Black American women in the U.S.

In summary, this integrative review of the evidence on lifestyle interventions targeting cardiometabolic health among Black American women of childbearing age revealed significant research gaps and areas for improvement. Key issues include nearly half of the studies having less than 70% representation of Black American women, a predominant focus on health education, insufficient attention to essential cardiometabolic outcomes (e.g., blood pressure and glycemic indices), and scarcity of research during the pre-pregnancy phase. While digital health technologies, such as mobile applications and social media, have shown promise, they remain underutilized in current interventions. Common weaknesses exist across studies, including small sample sizes, high attrition rates, and challenges in participant engagement, particularly among Black American women. These findings underscore the need for more comprehensive, culturally responsive, and technologically integrated approaches to address cardiometabolic health disparities in this population.

### Limitations

This review should be interpreted with recognition of some inherent limitations. First, while this study aimed to present evidence specifically for Black American women of childbearing age, several studies had diverse racial samples without separate analyses for Black American participants, with only a few studies focusing exclusively on this group. Second, the inclusion of only peer-reviewed English articles may have missed relevant evidence from non-English sources. Third, although lifestyle is influenced by structural factors that underlie cardiometabolic health disparities, this review did not evaluate the extent to which interventions addressed or impacted system-level determinants. Despite these constraints, this review highlights the limited studies addressing the cardiometabolic health of Black American women of childbearing age, a population particularly vulnerable to cardiometabolic diseases.

## Conclusions

This review provides preliminary evidence of lifestyle interventions’ potential impact on weight reduction, increased physical activity, healthier dietary behaviors, and decreased depression among Black American women of childbearing age. However, current evidence insufficiently addresses the full spectrum of cardiometabolic health challenges in this population. Future studies must adopt a more comprehensive approach by incorporating broader cardiometabolic outcomes and extending interventions to the pre-pregnancy period. Digital health technologies should be integrated more effectively into intervention strategies to improve accessibility and continuity of care, thus addressing recruitment, engagement and retention challenges. These improvements, along with more representative study populations and larger sample sizes, are crucial for developing culturally responsive interventions and generating rigorous evidence. Ultimately, such methodological advancements will foster equitable health solutions that truly meet the needs of Black American women of childbearing age, thereby reducing health disparities in this underserved population.

## Electronic supplementary material

Below is the link to the electronic supplementary material.

**Supplementary Material 1:** Supplementary Table 1

**Supplementary Material 2:** Supplementary Table 2

## Data Availability

All data generated or analyzed during this study are included in this published article [and its supplementary information files].

## Abbreviations

U.S.: United States
DM: Diabetes Mellitus
GDM: Gestational Diabetes Mellitus
NHWs: Non-Hispanic Whites
PA: Physical Activity
RCT: Randomized Controlled Trial
HbA1c: Glycated Hemoglobin
