# Supplementary material for: Lifestyle interventions addressing cardiometabolic health among Black American women of reproductive age in the U.S. : an integrative review
Source: BMC Pregnancy Childbirth. 2025 May 19;25:590. doi: 10.1186/s12884-025-07490-7 (PMC12090521; doi:10.1186/s12884-025-07490-7)
Supplement: Supplementary file 1 — Supplementary Material 1: Supplementary Table 1 [file 12884_2025_7490_MOESM1_ESM.docx]

Supplementary Table 1 Summary of Literature Search

| **Databases & Platforms** | **Date Performed** | **Search Strategies** | **Number of Results** |
| --- | --- | --- | --- |
| **PubMed (NLM/NCBI)** | 2023/06/12 | ("Black People"[Mesh] OR "african american*" OR black[tiab] african[tiab] OR blacks[tiab] OR "black american*"[tiab]) AND (((reproductive OR childbearing OR "child-bearing") AND age[tiab]) OR "Perinatal Care"[Mesh] OR "Postpartum Period"[Mesh:NoExp] OR Puerperium OR pre-pregnan* OR prepregnan* OR pre-natal* OR prenatal* OR post-partum OR postpartum OR perinatal OR peri-natal) AND ("Life Style"[Mesh] OR "Health Behavior"[Mesh] OR "Body Weight"[Mesh:NoExp] OR "Diet Therapy"[Mesh] OR "Mindfulness"[Mesh] OR "Exercise"[Mesh] OR "Psychosocial Intervention"[Mesh] OR "Self-Management"[Mesh] OR "Self Care"[Mesh:NoExp] OR "Sleep"[Mesh] OR "Stress, Psychological/prevention and control"[Mesh] OR lifestyle OR "life style" OR "health coach*" OR "weight manage*" OR "Weight Control" OR diet* modif* OR "physical activity" OR "self care" OR "self manag*" OR (health* AND (behavior* OR behaviour*)) OR mindfullness OR mindfulness OR sleep[tiab] OR "stress manag*") AND ("Cardiometabolic Risk Factors"[Mesh] OR "Diabetes, Gestational"[Mesh] OR "Hypertension, Pregnancy-Induced"[Mesh] OR "Blood Pressure"[Mesh] OR "Blood Glucose"[Mesh] OR "Glycated Hemoglobin"[Mesh] OR "Glycemic Control"[Mesh] OR "Body Mass Index"[Mesh] OR "Heart Rate"[Mesh] OR "Inflammation"[Mesh] OR "Cytokines"[Mesh] OR "Psychosocial Functioning"[Mesh] OR "Social Support"[Mesh] OR "Quality of Life"[Mesh] OR "Self Efficacy"[Mesh] OR "Sleep Wake Disorders"[Mesh] OR "Psychological Distress"[Mesh] OR "Depression"[Mesh] OR "Anxiety"[Mesh:NoExp] OR "Stress, Psychological"[Mesh] OR "Health Literacy"[Mesh] OR cardio-metabolic OR cardiometabolic OR gestational diabetes OR gestational hypertension OR pre-eclampsia OR preeclampsia OR "blood pressure" OR diastolic OR systolic OR blood glucose OR A1c OR "Hemoglobin A1c" OR HbA1c OR "Hb A1c" OR glycemia OR glycemic control OR "lipid profile*" OR "body mass index" OR BMI[tiab] OR "Heart Rate Variability" OR "heart rate*" OR inflammation OR cytokine* OR interleukin OR psycho-social OR psychosocial OR "social support" OR "quality of life" OR QOL[tiab] OR "self efficacy" OR "sleep intervention*" OR sleep disturb* OR sleep duration OR sleep parameters OR sleep regularity OR insomnia* OR dyssomnia* OR distress OR depression OR anxiety OR stress[tiab] OR "health litera*") AND ("Clinical Study" [Publication Type:NoExp] OR "Clinical Trial" [Publication Type] OR "Clinical Trial Protocol" [Publication Type] OR "Pilot Projects"[Mesh] OR "clinical trial*" OR "controlled trial*" OR experimental intervention* OR quasi-experimental OR quasiexperimental OR pilot intervention OR pilot study OR feasibility intervention OR feasibility stud* OR random*[tiab] OR RCT[tiab])  Filters applied: English | 140 |
| **CINAHL Complete (EBSCOhost)** | 2023/06/12 | (MH "Black Persons+") OR "african american*" OR (black N3 african*) OR blacks OR (black N3 american*)  AND  (MH "Childbearing Age") OR ((reproductive OR childbearing OR "child-bearing") N3 age) OR (MH "Postnatal Period") OR (MH "Postpartum (Omaha)") OR pre-pregnan* OR prepregnan* OR pre-natal* OR prenatal* OR post-partum OR postpartum  AND  (MH "Life Style Changes") OR (MH "Life Style") OR (MH "Weight Control") OR (MH "Weight Management (Iowa NIC)") OR (MH "Diet Therapy+") OR (MH "Health Behavior") OR (MH "Mindfulness") OR (MH "Physical Activity (Omaha)") OR (MH "Physical Activity") OR (MH "Psychosocial Care (Saba CCC)") OR (MH "Psychosocial Intervention") OR (MH "Self-Management") OR (MH "Self Care") OR (MH "Sleep") OR (MH "Stress Management") OR lifestyle OR "life style" OR "health coach*" OR "weight manage*" OR (diet* N3 modif*) OR "physical activity" OR "self care" OR "self manag*" OR (health* N3 (behavior* OR behaviour*)) OR mindfullness OR mindfulness OR TI sleep OR AB sleep OR (stress* N3 manage*)  AND  (MH "Cardiometabolic Risk Factors") OR (MH "Diabetes Mellitus, Gestational") OR (MH "Pregnancy-Induced Hypertension+") OR (MH "Blood Pressure") OR (MH "Systolic Pressure") OR (MH "Diastolic Pressure" OR (MH "Blood Glucose") OR (MH "Glycated Hemoglobin") OR (MH "Glycemic Control") OR (MH "Body Mass Index") OR (MH "Heart Rate Variability") OR (MH "Heart Rate") OR (MH "Inflammation") OR (MH "Cytokines") OR (MH "Interleukins") OR (MH "Psychosocial Adjustment: Life Change (Iowa NOC)") OR (MH "Psychosocial Aspects of Illness+") OR (MH "Psychosocial Functioning") OR (MH "Psychosocial Health (Iowa NOC)+") OR (MH "Support, Social+") OR (MH "Social Support (Iowa NOC)") OR (MH "Social Support Index") OR (MH "Support, Psychosocial+") OR (MH "Quality of Life (Iowa NOC)") OR (MH "Quality of Life") OR (MH "Quality of Working Life") OR (MH "Health and Life Quality (Iowa NOC)") OR (MH "Self-Efficacy") OR (MH "Sleep Deprivation") OR (MH "Sleep Disorders") OR (MH "Sleep Duration") OR (MH "Sleep Hygiene") OR (MH "Sleep Latency") OR (MH "Sleep Quality") OR (MH "Insomnia+") OR (MH "Dyssomnias") OR (MH "Psychological Distress") OR (MH "Depression") OR (MH "Anxiety") OR (MH "Stress, Psychological") OR (MH "Health Literacy") OR cardio-metabolic OR cardiometabolic OR gestational diabetes OR "gestational hypertension" OR pre-eclampsia OR preeclampsia OR "blood pressure" OR "Hb A1c" OR HbA1c OR A1c OR blood glucose OR glycemia OR glycemic control OR "Hemoglobin A1c" OR "lipid profile*" OR "body mass index" OR BMI OR "heart rate*" OR ("heart rate*" N3 variabil*) OR inflammation OR cytokine* OR interleukin* OR dyssomnia* OR insomnia* OR psycho-social OR psychosocial OR (social N3 support*) OR "quality of life" OR TI QOL OR AB QOL OR "self efficacy" OR (sleep N3 (quality OR duration OR parameters OR regularity)) OR (sleep N3 disturb*) OR "sleep intervention*" OR insomnia* OR distress OR anxiety OR stress* OR TI depression OR AB depression OR "health litera*"  AND  PT Randomized Controlled Trials OR TI random* OR AB random* OR PT Clinical Trial OR "clinical trial*" OR "controlled trial*" OR experimental intervention* OR quasi-experimental OR quasiexperimental OR pilot intervention OR pilot study OR feasibility intervention OR feasibility stud*  Limiters - English Language  Expanders - Apply equivalent subjects  Search modes - Boolean/Phrase | 65 |
| **Web of Science Core Collection (Clarivate)** | 2023/08/02 | #1 - TS=(black NEAR/3 (people OR person* OR population* OR patient*)) OR TS="african american*" OR TI="black african*" OR AB="black african*" OR TI=blacks OR AB=blacks OR TI="black american*" OR AB="black american*"  AND  #2 - TS=(((reproductive OR childbearing OR "child-bearing") NEAR/3 age) OR "Perinatal Care" OR "Postpartum Period" OR Puerperium OR pre-pregnan* OR prepregnan* OR pre-natal* OR prenatal* OR post-partum OR postpartum OR perinatal OR peri-natal)  AND  #3 - TS=((psychosocial NEAR/3 intervention*) OR lifestyle OR "life style" OR "health coach*" OR "body weight" OR "diet therap*" OR "weight manage*" OR "weight control" OR (diet* NEAR/3 modif*) OR exercise OR "physical activity" OR "self care" OR "self manag*" OR (health* NEAR/3 (behavior* OR behaviour*)) OR mindfullness OR mindfulness OR sleep OR "stress manag*")  AND  #4 - TS=(cardio-metabolic OR cardiometabolic OR gestational diabetes OR gestational hypertension OR pre-eclampsia OR preeclampsia OR "blood pressure" OR diastolic OR systolic OR blood glucose OR A1c OR "Hemoglobin A1c" OR HbA1c OR "Hb A1c" OR "glycated hemoglobin" OR glycemia OR glycemic control OR "lipid profile*" OR "body mass index" OR BMI OR "Heart Rate Variability" OR "heart rate*" OR inflammation OR cytokine* OR interleukin OR psycho-social OR psychosocial OR "social support" OR "quality of life" OR QOL OR "self efficacy" OR (sleep NEAR/3 (intervention* OR disturb* OR duration OR parameters OR regularity)) OR insomnia* OR dyssomnia* OR distress OR depression OR anxiety OR stress OR "health litera*")  AND  #5 - TS=("Pilot Project*" OR "clinical trial*" OR "clinical study" OR "controlled trial*" OR experimental intervention* OR quasi-experimental OR quasiexperimental OR pilot intervention OR pilot study OR feasibility intervention OR feasibility stud* OR random*)  #5 AND #4 AND #3 AND #2 AND #1 and English (Languages) and Editorial Material or Book Chapters (Exclude – Document Types) | 120 |
| **Scopus (Elsevier)** | 2023/08/02 | ( TITLE-ABS-KEY ( ( black W/3 ( people OR person* OR population* OR patient* ) ) ) OR TITLE-ABS-KEY ( "african american*" ) OR TITLE ( "black african*" ) OR ABS ( "black african*" ) OR TITLE ( blacks ) OR ABS ( blacks ) OR TITLE ( "black american*" ) OR ABS ( "black american*" ) ) AND ( TITLE-ABS-KEY ( ( ( reproductive OR childbearing OR "child-bearing" ) W/3 age ) OR "Perinatal Care" OR "Postpartum Period" OR puerperium OR pre-pregnan* OR prepregnan* OR pre-natal* OR prenatal* OR post-partum OR postpartum OR perinatal OR peri-natal ) ) AND ( TITLE-ABS-KEY ( ( psychosocial W/3 intervention* ) OR lifestyle OR "life style" OR "health coach*" OR "body weight" OR "diet therap*" OR "weight manage*" OR "weight control" OR ( diet* W/3 modif* ) OR exercise OR "physical activity" OR "self care" OR "self manag*" OR ( health* W/3 ( behavior* OR behaviour* ) ) OR mindfullness OR mindfulness OR sleep OR "stress manag*" ) ) AND ( TITLE-ABS-KEY ( cardio-metabolic OR cardiometabolic OR gestational AND diabetes OR gestational AND hypertension OR pre-eclampsia OR preeclampsia OR "blood pressure" OR diastolic OR systolic OR blood AND glucose OR a1c OR "Hemoglobin A1c" OR hba1c OR "Hb A1c" OR "glycated hemoglobin" OR glycemia OR glycemic AND control OR "lipid profile*" OR "body mass index" OR bmi OR "Heart Rate Variability" OR "heart rate*" OR inflammation OR cytokine* OR interleukin OR psycho-social OR psychosocial OR "social support" OR "quality of life" OR qol OR "self efficacy" OR ( sleep W/3 ( intervention* OR disturb* OR duration OR parameters OR regularity ) ) OR insomnia* OR dyssomnia* OR distress OR depression OR anxiety OR stress OR "health litera*" ) ) AND ( EXCLUDE ( DOCTYPE , "le" ) ) AND ( LIMIT-TO ( LANGUAGE , "English" ) ) | 27 |
