# Supplementary material for: Lifestyle interventions addressing cardiometabolic health among Black American women of reproductive age in the U.S. : an integrative review
Source: BMC Pregnancy Childbirth. 2025 May 19;25:590. doi: 10.1186/s12884-025-07490-7 (PMC12090521; doi:10.1186/s12884-025-07490-7)
Supplement: Supplementary file 2 — Supplementary Material 2: Supplementary Table 2 [file 12884_2025_7490_MOESM2_ESM.pdf]

Supplementary Materials

Table 2. Characteristics of Studies

Authors	Study Design	Location	Participants	Stage	Sample Size	% of Black/ African American Participants	Intervention and Delivery Approach	Dose of Intervention
Thompson et al., 2016 [48]	RCT<br>I: PATE (Lifestyle-enhanced PAT home visits)<br>C: PAT (Usual Control)	Washington, Bolivar, or Humphreys County in Mississippi	N=82 (PATE: 39 vs. PAT: 43), predominantly low-income pregnant women who were overweight and obese; mean age 22.7 ±4.69 (PATE) vs. 23.3 ± 4.58 (PAT)	Pregnancy	82	95%	**PATE intervention (PATE)**: healthy eating, physical activity (e.g., goal setting, instructional DVDs); **Delivered** by trained parent educators.	90 to 120 min (PATE)/month x 6 months
Thomson et al. 2018 [50]	RCT<br>I: PATE (Lifestyle-enhanced PAT home visits)<br>C: PAT (Usual Control)	Washington, Bolivar, or Humphreys County in Mississippi	N=54 (I: 24 vs. C: 30) postpartum women from rural areas, PATE mean postpartum BMI for control: 30.4, for intervention group: 31.6	Postpartu m	54	95%	**PATE intervention (PATE)**: culturally tailored maternal weight management (e.g., set exercise goals, instructional DVDs), early childhood obesity prevention, home visits, optional monthly group meetings, developmental screening, and resource network; **Delivered** by trained parent educators.	Up to 12 home visits for 18 months (6 months pregnancy and 12 months postpartum)
Berry et al., 2015 [23]	RCT<br>I: Weight management intervention<br>C: Control group	WakeMed Health & Hospitals and UNC REX Healthcare in Raleigh, North Carolina	N=60 (I=30 vs. C:30) postpartum women (≥ 6 weeks postpartum); mean age: 27.2 ± 6.4	Postpartu m	60	77%	**Weight Management**: group class education on nutrition, exercise (a pedometer), and coping skills for metabolic control and nutrition; **Delivered** by health educators.	60-minute/class x 12 classes and 3 monthly follow-ups

Boyd et al 2019 [25]	RCT<br>I: Parents Interacting with Infants (PIWI) delivered via social media<br>C: PIWI delivered in-person	Urban pediatric primary care clinics within a larger pediatric care network, the Children's Hospital of Philadelphia, PA	N=24 (I:12 vs. C: 12) postpartum women with postpartum depression from unspecified pediatric clinic; Intervention mean age: 26.4 ± 1.9 vs. Control mean age: 26.3 ± 1.8	Postpartum	24	84%	**Postpartum Depression and Parenting (PIWI):** parenting program including 8 topics (depression education, infant temperament, play, feeding, safety, sleep, parent-child interactions, and reading; **Delivered** by facilitator via social media.	1-2 hrs/wk x 8 weeks
Crockett et al 2008 [28]	RCT<br>I: ROSE intervention<br>C: Treatment as usual	Hospital-affiliated prenatal clinic in the Mississippi Delta	N=36 (I: 19 vs.C:17) low-income pregnant women; mean age: 23.4±4.98	Pregnancy and postpartum	36	100%	**Depression Prevention (ROSE)**: involving individual and group sessions on coping and depression prevention; **Delivered** by therapist.	Pregnancy: 90 min/week x 4 weeks<br>Postpartum: 50 min/week x 2 weeks
Berry et al., 2016 [24]	RCT cluster<br>I: Group Perinatal Health Classes<br>C: Wait-List	WakeMed Health & Hospitals and UNC REX Healthcare in Raleigh, North Carolina	N= 100 (I:50 vs. C=50); pregnant women (22-36wks) with a diagnosis of GDM; mean age: 30.16±6.26	Pregnancy and postpartum	100	52%	**Group Perinatal Health Class:** education on the benefits of breastfeeding for metabolic control, nutrition, and exercise; **Delivered** by interventionists and received monthly motivational text messages	60minutes/class x 14 weekly classes from pregnancy to postpartum and 3 monthly postpartum classes
Joshi et al. 2018 [31]	RCT:<br>I: Revolutionizing Exercise and Nutrition Everyday in Women (RENEW)<br>C: Usual care	Boston Medical Center	N=62 (I: 32 vs. C:32) postpartum women; Intervention mean age: 30.2 ± 6.1 vs. Control mean age: 31.2 ± 6.3; Intervention mean BMI: 35.6 ± 7.7 vs. Control BMI: 34.4 ± 5.3	Postpartum	62	100%	**Weight Management (RENEW):** in-person weight loss involving nutrition education and physical activity (resistance exercise and dancing); **Delivered** by the dietitian and peer health worker.	~35 min or more/week x 8 weeks

Liu et al 2015 [54]	RCT Pilot<br>I: Lifestyle intervention<br>C: Control with medical data retrieved from the medical chart	South Carolina	N=16 (I: 16 vs. C: 38), overweight or obese African American women at 13.2±2.5 weeks of gestation; mean age 25.1 ± 4.2 years	Pregnancy and postpartu m	16	100%	**Lifestyle intervention:** self-monitoring of physical activity (pedometer), diet, and weight (scale)self, how to self-regulate (e.g., set goals, problem solve) and cope in high-risk situations; **Delivered** by a registered dietitian and an African American research staff member.	Pregnancy: 1 individual counseling session followed by 8 group sessions alternated with telephone counseling contacts.<br><br>Postpartum: received a home visit and up to three counseling calls to promote postpartum weight loss through week 12.
Walker et al. 2012 [51]	RCT<br>I: Weight retention<br>C: Wait-list	Texas	N= 71 (I: 34 vs. C: 37) overweight postpartum low-income women; mean age: 24.6 ± 4.8; mean BMI: 33.5 ± 6.4	Postpartu m	71	35%	**Ethnic tailored Weight Management**: increase self-efficacy related to eating patterns, weight (digital scale), physical activity (pedometers), stress, and psychosocial well-being; **Delivered** by RNs and health educators.	2 hrs/wk x 13 weeks.
Liu et al 2022 [42]	RCT<br>I: Behavioral lifestyle intervention<br>C: Standard care	South Carolina	N=228 (I: 114 vs. C: 114) pregnant women (≤16 wks) overweight and obesity; pre-pregnancy BMI: 32.3 ± 5.9 .6% had obesity pre-pregnancy); mean age: 29.7+5.0	Pregnancy and postpartu m	228	44%	**Postpartum Weight management:** feedback on diet and physical activity (pedometer), personalized weight gain (scale) tracking graphs, brief telephone counseling, behavioral podcast, social media	Pregnancy: 1 in-depth counseling before or at 18 gestational weeks; 10 weekly brief telephone counseling and

							support, and weight monitoring; **Delivered** by interventionists.	accompanying podcasts.<br><br>Postpartum: 1 in-depth counseling at 6-8 weeks; biweekly counseling calls for 6 months.
Wilcox et al. 2022 [49]	RCT<br>I: Lifestyle intervention<br>C: Standard care	South Carolina	N=219 (I=112 vs. C: 107) pregnant women , overweight and obesity mean age: I 30.4 ±5.2 vs. C: 29.1 ± 4.8; Pre pregnancy BMI: I 33 ± 6.6 vs. C 33.9 ± 6.1	Pregnancy	219	44%	**Behavioral Intervention**: diet and physical activity self-monitoring (Sense Wear Armband) and counseling calls with behavioral podcasts and use of a private Facebook group; **Social support**: identified a peer to support behavior change. **Delivered** by staff training in public health and behavior change.	10 weekly counseling calls and 10 corresponding behavioral podcasts
Gross et al. 2018 [30]	RCT I: Integrated NIP and Enhanced Women, Infant and Children Special Supplemental Nutrition Program<br>C: Usual care	Johns Hopkins Women Infants and Children's Program (WIC) from the Johns Hopkins Hospital Outpatient Center's Nutrition in Pregnancy Clinic (NIP), Maryland	N=53 (I: 23 vs. C: 30) pregnant low-income women; mean age: 26.1± 4.4; mean pre-pregnancy BMI: 39.9±7.6	Postpartu m	53	96%	**Postpartum Weight Retention:** enhanced nutrition service on weight regulation, exercise, and behavior change strategies using brief videos, and print materials; **Delivered** by a registered dietitian.	5 healthy eating classes, 1 telephone counseling,<br>2 text messages/wk up to 6 months postpartum

Liu et al 2021[43]	RCT<br>I: Behavioral lifestyle intervention<br>C: Standard care	South Carolina	N=228 (I: 114 vs. C: 114) pregnant women (≤16 wks) , overweight and obesity; pre-pregnancy BMI: 32.3 ± 5.9 (51.6% obese pre-pregnancy); mean age: 29.7+5.0	Pregnancy and postpartum	228	44%	**Weight Management:** self-monitoring weight (scale), physical activity (Sense Wear Armband), dietary practices. **Social support:** all participants join a Facebook. **Delivered** by interventionists.	1 in-depth counseling at ≤ 18 weeks of gestation<br>10 weekly individual phone calls followed by weekly or biweekly counseling calls
El-Mohandes et al., 2008 [29]	RCT I: Reduction of psychosocial risks in postpartum (smoking, ETSE, depression, IPV)<br>C: Usual care	Washington, DC	N=913 (I: 452 vs.C: 461) pregnant women, mean age: 24±0.3	Pregnancy and postpartum	913	100%	**Reduction of Psychosocial Risks:** prenatal clinic-based individualized counseling based on the transtheoretical stage of risky health behaviors; **Delivered** by social work and mental health professionals.	Pregnancy: 45 min/sessions x 4-8 sessions; Postpartum: 45 min/sessions x up to 2 sessions
Grote et al 2009 [32]	RCT<br>I: Enhanced interpersonal psychotherapy (IPT-B)<br>C: Enhanced usual care	Women's Hospital in Pittsburgh, Pennsylvania	N=53 (I: 25 vs. C: 28); gestational week: 22.6±6.7; low-income ; Edinburgh Postnatal Depression Scale (>12); mean age: 24.3 ± 5.3	Pregnancy	53	62%	**Depression Psychotherapy (IPT-B):** a multicomponent model of care, including an engagement session, acute IPT-B resolving interpersonal problem areas (role transition, role dispute, grief, and interpersonal deficits), and maintenance IPT; **Delivered** by doctoral (psychologist) and master students (social work). If needed, phone sessions were available.	Weekly IPT-B session/ x 8 weeks<br>Maintain phase: biweekly or monthly up to six months postpartum

Cinciripini et al., 2020 [27]	RCT<br>I: Cognitive behavioral analysis system of psychotherapy (CBASP)<br>C: Matched control focused on health and wellness (HW)	University of Texas M. D. Anderson Cancer Center	N= 157 (I: 128 vs.C: 129) pregnant women, mostly low-income; mean age: 25±5.9	Pregnancy	157	54%	**Depression-focused Intervention for Smoking Cessation (CBASP):** in-person counseling based on cognitive behavioral analysis system of psychotherapy; **Delivered** by Ph.D. therapists.	60 min/session x 10 sessions
Kranzler et al.2021 [38]	RCT<br>I: Bupropion<br>C: Placebo	Philadelphia, PA	N = 129 (I: 64 vs. C:65), low-income, pregnant mothers (13-26 weeks of gestation), current smokers; mean age: 28.5 ± 4.8	Pregnancy and postpartu m	129	52%	**Medication Intervention:** bupropion given with smoking cessation counseling sessions; **Delivered** by staff via text messaging system.	10 weeks of given medication<br>6 counseling sessions: 4 during pregnancy & 2 postpartum
McKee et al 2006 [44]	RCT<br>Three arms (depression intervention vs. treatment as usual vs. non-depressed group)	Community health centers in the south Bronx of New York City	N= 187 (I: 100 vs. C87) low-income minority Black and Hispanic pregnant women recruited from community health centers, 100 with elevated depression (53 intervention vs. 47 TAU) and 87 non-depressed comparison group. Mean age: 24.7± 5.5; gestational age at enrollment: 28.4 ± 2.5; 19.3% married.	Pregnancy and postpartu m	187	43%	**Depression Intervention**: 1) CBT developed for patients in primary care (e.g., cognitive distortion and reframing, relaxation technique, 44<br>setting) 2) child-development (CD) psychoeducation modules and 3) social support building (SSB) activities; **Delivered** in person or by telephone.	8 CBT and education sessions
Chao et al. 2017 [26]	RCT<br>I: Telephone counseling & mhealth weighing<br>C: Treatment as usual	Obstetrics clinics in Philadelphia	N=41 (1: 20 vs.C:21) pregnant mothers, mean age: 28.7±5.8 years; mean pre-pregnancy BMI: 31.2 ± 6.2 kg/m2	Pregnancy	41	54%	**Weight Management:** telephone counseling on behaviors related to weight loss and mhealth weighing; **Delivered** by a dietitian.	20-min telephone counseling/wk between 16-36 weeks of pregnancy

South et al. 2021 [47]	RCT I: Natured Connect & Education C: Attention control (Education session)	Philadelphia, PA	N= 36 (I: 17 vs. C: 19) postpartum women from lower-resourced, minority neighborhoods; mean age: 28±6; mean BMI: 32.5 ± 4.3	Postpartum	39	90%	**Nature Connect**: spending time in nature with a nature coach who performed a home visit on day 1, a park visit on day 8, and a phone call check-in on day 15; personalized reminders and feedback were received through texts; **Delivered** by a nature coach of community health experience	1 hr/wk x 4 weeks
Jesse et al 2015 [36]	RCT I: Insight-Plus+Cognitive Behavioral Intervention C: Usual care	Local health department and regional perinatal center, East Carolina	N=146 (I: 72 vs. C: 74) rural, minority, low-income pregnant women with Edinburgh Postnatal Depression Scale score > 4; mean age: 25.05±5.49	Pregnancy	146	68%	**Depression Intervention (Insight-Plus+Cognitive Behavioral Intervention)**: psychoeducation of perinatal depression, coping, relaxing, domestic violence, grief loss, spirituality; MP3 player for stress reduction; **Delivered** by a clinical social worker, other licensed mental health professionals, and a “resource mom” (a paraprofessional staff member).	6 group sessions: 2 hrs/group per week with weekly booster sessions over telephone.
Haire-Joshu et al. 2019 [33]	RCT I: Parents as Teachers (PAT) + Lifestyle C: Usual care (PAT)	Barnes-Jewish Hospital and Washington University School of Medicine in St. Louis, Missouri	N=185 (I: 92 vs. C: 93) socioeconomic disadvantaged background; obese and overweight at pregnancy onset; Intervention mean age: 24.7 ± 5.3 vs. Control mean age: 24.7±5.6	Pregnancy and postpartum	185	100%	**Weight Management:** reinforced eating and activity behaviors learned prenatally while also addressing challenges faced by the new parent (e.g., infant feeding, sleep deprivation); **Delivered** by parent educators.	Pregnancy: bi-weekly home visit<br><br>Postpartum: monthly home visit until 12 months postpartum

Hans et al., 2018 [34]	RCT I: Doula-home-visitation program C: Case Management Control	High-poverty Illinois communities	N=312 (I:156 vs.C:156); pregnant women, Intervention mean age: 18.5 ± 2.0 vs. Control mean age 18.3± 1.6	Pregnancy and postpartu m	312	45%	**Doula combined Home Visitation:** doula provided perinatal health education (e.g., prenatal classes; pregnancy health, childbirth preparation, breastfeeding, newborn care, postpartum health), occasionally attended medical visits, attended hospital births; home visitors focused on building mother-infant relationship, child development, child safety, and educational-work planning, screening for family basic needs; **Delivered** by doulas and home visitors.	Pregnancy-1st week postpartum: weekly home visit primarily by doula<br><br>6 weeks – 3 months Postpartum: weekly session primarily by home visitor
Kitzman et al. 1997 [37]	RCT I (T3 & T4): Family Nurse Home Visitation C (T1 & T2): Attention control	Obstetrical clinic at the Reginal Medical Center in Memphis	N= 1,139 (I: 458 vs. C: 681) primarily African-American women at less than 29 weeks' gestation, with no previous live births, and with at least 2 sociodemographic risk characteristics (unmarried, <12 years of education, unemployed). Mean age: 18 years (SDs reported for T1-T4)	Pregnancy and 24 months postpartu m	1,139	90%	**Family Nurse Home Visitation**: health education about signs/symptoms of pregnancy complications, parenting, child development; provide social support and assistance with life-course development (pregnancy planning, educational achievement, employment); **Delivered** by nurse.	Pregnancy: ~7 home visits<br><br>Birth - 24 months postpartum: ~26 home visits

Herring et al. 2014 [35]	RCT pilot<br>I: Healthy4Baby<br>C: Usual care	Two large outpatient practices which served primarily Medicaid-insured patients in Philadelphia, PA.	N = 18 (I: 9 vs. C: 9) low-income (all on Medicaid), obese urban postpartum mothers; mean age: 24.2 ± 5.1, BMI: 36.9 ± 6.1	Postpartum	18	78%	**Weight Management (Healthy4Baby):** limit sugary drinks, junk, and high-fat foods with a caloric goal; increase physical activity (pedometer) and hours of sleep, self-weighing (digital scale) goal tracking. **Social support**: all participants joined the Facebook<br>**Delivered** via text messaging and Facebook.	15min/tele-health coaching biweekly x 14 weeks<br><br>self-monitoring texts 3 to 4 times weekly
Lane et al 2023 [40]	RCT<br>I: Lifestyle counseling<br>C: Usual care	Southeastern US	N=219 (I: 112 vs. C: 107) pregnant and postpartum women with pre-pregnancy overweight or obesity; mean age: 29.7± 0.3	Pregnancy and postpartum	219	44%	**Weight Management:** Lifestyle counseling involving dietary and physical activity counseling related to gestational weight gain and postpartum weight retention through educational handouts, podcasts, self-monitoring (pedometers, scales); **Social Support**: all participants joined a private Facebook group.<br>**Delivered** by trained interventionists.	In-depth counseling before week 18 of pregnancy and early postpartum respectively;<br><br>Weekly or biweekly telephone counseling after the 1st in-depth counseling

Napolitano et al 2021 [45]	RCT I: BeFAB virtual coaching<br>C: Usual care	Washington, DC	N = 136 (I: 65 vs. C: 71); postpartum mothers, mean age: 27.8 ± 5.4; mean BMI = 32.5 ± 4.3	Postpartum	136	100%	**Digital Lifestyle Health Coaching**: virtual coaching on meal preparation, managing cravings, physical activity tips, and goal setting. **Social Support**: all participants join the Facebook. **Delivered** using a private Facebook group and an app (BeFAB).	5 days/wk x 12 weeks
Lewey et al., 2022 [41]	RCT I: Text-Based Gamification Intervention<br>C: Attention control	Penn Medicine hospitals, Philadelphia, PA.	N= 127 (I: 64 vs. C: 63) postpartum mothers, 52% on Medicaid insurance, 30% taking BP medications, mean age32± 5.6; mean BMI 32±7.5	Postpartum	127	55%	**Physical Activity:** Use of gamification; points and acquired levels for daily step goal achievement. **Social support**: team-based gamification. **Delivered** digitally using a wearable device and receiving text messages	12 weeks
Pezley et al., 2022 [46]	Randomized pilot Two active treatments I1: Sunnyside vs. I2: Sunnyside Plus	Participants from a national pregnancy digital platform	N=22 (I1=9 vs. I2:13) pregnant mothers with 64% having obesity; mean age 30.4 ± 3.9	Pregnancy & Postpartum	22	100%	**CBT intervention**: Web-based education and text messages targeting skills to manage mood and breastfeeding education for 6 weeks during pregnancy and up to 6 weeks postpartum. **Delivered** by lactation specialists	Pregnancy: 40-60 min/wk x 6 weeks; Postpartum: 10-20 min/wk x 6 weeks
Bryant et al. 2023 [52]	Quasi-experimental (pre-post) without control group<br>I: Joyuus, a mobile program	Participants were from 27 states in the U.S.	N=87 pregnant mothers; mean age: 30 years (SD not reported)	Pregnancy and postpartum	87	99%	**Self-Care App (Joyuus)**: health education through a self-care app to address psychosocial issues. **Delivered** digitally.	Used Joyuus for one month

Cavallo et al. 2016 [55]	Quasi-experimental (pre-post) without control group<br>I: Web-based weight loss intervention	Family planning clinic located at a health department, North Carolina	N=40 low-income women of childbearing age; mean age: 30± 6.5; mean BMI (kg/m^2): 39±8.5; 83% with obesity, 25% with HTN, 10% with DM, 10% with high cholesterol	Pre-conception	40	73%	**Weight Management**: group-based face-to-face education and web-based modalities focusing on physical activity, and nutrition; **Delivered** digitally.n	70-120 min/wk x 20 weeks
Kannan, 2010 [53]	Pilot study with pre-and post-test without control group<br>I: Healthy Eating and Harambee intervention	Urban community centers in Genessee Country, Michigan.	N=102 healthy, low income, non-pregnant African-American women; mean age = 27.5 (SD not reported)	Pre-conception	102	100%	**Lifestyle Intervention (Healthy Eating and Harambee):**<br>Healthy eating habits, nutrition, meal preparation, biomedical self-monitoring (Omron BP cuff), body image; **Delivered** by peers.	1 session per wk/ x 13 weeks
Krukowski et al 2021 [39]	RCT: 2x2x2 factorial design including 8 groups	Obstetric clinic, Memphis, Tennessee	N=40 pregnant women in their first trimester, 56% with overweight/obesity; mean age: 29.1 ± 12.5	Pregnancy	40	62%	**Incentive Intervention**: incentives for self-weighing on a digital scale, incentives for following the GWG guidelines, incentives for meeting physical activity goals; **Delivered** digitally using emails, text messages, wireless scales,	Participants' daily self-weighing behavior and weekly physical activity (measured in active minutes)/ for 6 months
